# Influence of Local and Reimported United States and South American Corn Sources on Broiler Performance, Nutrient Digestibility, and Processing Yield

**DOI:** 10.3390/ani15121770

**Published:** 2025-06-16

**Authors:** Maria J. Brizuela, Jose I. Vargas, Isabella C. Dias, Joseph P. Gulizia, Eva G. Guzmán, Jose R. Hernández, Cristina T. Simões, Wilmer J. Pacheco

**Affiliations:** 1 Department of Poultry Science, Auburn University, Auburn, AL 36849, USA; mzb0257@auburn.edu (M.J.B.); jiv0001@auburn.edu (J.I.V.); jzg0120@auburn.edu (J.P.G.); egg0035@auburn.edu (E.G.G.); jzh0237@auburn.edu (J.R.H.); crists02@gmail.com (C.T.S.); 2 Department of Animal Science, Federal University of Paraná, Curitiba 80035-050, PR, Brazil; isabelladias@ufpr.br

**Keywords:** corn origin, broiler performance, nutrient digestibility, processing yield

## Abstract

The origin of feed ingredients, particularly corn, can influence nutrient composition and availability, thereby affecting broiler growth performance and overall production efficiency. This study evaluated the effects of the dietary inclusion of different corn origins: United States (local) (USA-L), United States (reimported) (USA-R), Argentina (ARG), and Brazil (BRA) on broiler performance, nutrient digestibility, and processing yield from 1 to 35 d of age. A total of 1200 male broiler chicks (YPM × Ross 708) were randomly assigned to four dietary treatments, with each diet incorporating corn from a specific origin. Birds were housed in controlled environmental conditions and fed isocaloric, isoproteic diets formulated based on corn nutrient profiles from each origin. Body weight (BW), body weight gain (BWG), feed intake (FI), and feed conversion ratio (FCR) were assessed at 10, 21, and 35 d. On d 35, ileal digestibility of nutrients was assessed using titanium dioxide as an indigestible marker, and processing yields were measured on d 36. Results indicated no impact of corn origin on BW or BWG during the grow-out. However, FI was greater in broilers fed with diets containing corn from ARG and BRA compared to corn from USA-L from 1 to 35 d (*p* = 0.012). Feed intake of birds fed diets with USA-R did not differ from diets with either BRA or USA-L corn. Feed conversion ratio remained unaffected at 10 and 21 d of age, but broilers fed diets with corn from USA-L and USA-R exhibited improved FCR at 35 d compared to those fed corn from BRA (*p* < 0.001). Processing weights and yields showed no differences among treatments; nonetheless, broilers fed corn from ARG had a higher chilled carcass weight than those fed corn from USA-R (*p* = 0.032). Nutrient digestibility analysis revealed no differences in crude protein, fat, calcium, and potassium digestibility, while phosphorus digestibility was significantly higher in broilers fed corn from ARG compared to corn from USA-L (*p* = 0.007). These findings suggest that corn origin minimally affected overall broiler growth performance and carcass characteristics. However, differences in FI, FCR, and nutrient digestibility may exist among different corn sources.

## Simple Summary

Corn is an important ingredient in broiler diets, but its nutritional quality varies based on its origin. This study investigated how corn from the United States (local and reimported), Argentina, and Brazil affected the growth, nutrient digestibility, and processing yield of broilers from 1 to 35 days of age. Overall, corn origin had no effect on broiler growth and carcass traits. However, broilers fed diets with Argentinian and Brazilian corn had a higher feed intake, while those fed diets with corn from the USA (both local and reimported) had improved FCR. Broilers that consumed diets with Argentinian corn had improved phosphorus digestibility. Overall, corn origin had a minimal effect on broiler performance and processing yield, though differences in feed intake, FCR, and phosphorus digestibility were observed. Understanding these variations can help broiler producers make informed decisions to optimize diets, improving both sustainability and economic efficiency.

## 1. Introduction

Corn plays an important role in broiler diets due to its high energy contribution, which makes it an essential ingredient for optimal development and productive performance of broilers [1]. On average, corn provides around 3378 kcal/kg ME [2], which represents around 65% of the energy content of a typical broiler diet [3]. Most of the energy value of corn comes from its high amount of digestible starch. Additionally, corn is rich in essential amino acids such as glutamine, leucine, and alanine [4]; fat-soluble vitamins such as A, E, and K [5]; and minerals such as calcium, magnesium, phosphorus, potassium, sodium, and zinc [5], which support broiler health and immune function [6].

Corn composition varies in terms of starch, oil, protein, and non-starch polysaccharides. Genetic differences influence nutrient composition, as some varieties are bred for higher starch, protein, or oil content [7]. Agronomic conditions, including soil quality, climate, fertilization practices, and irrigation, further impact nutrient deposition in the corn kernel by influencing plant metabolism and nutrient uptake [8]. Likewise, post-harvest handling and storage conditions can alter moisture content and promote the degradation of key components such as protein, amino acids, fatty acids, and vitamins, or increase the activity of mycotoxin-producing fungi [9,10,11]. Understanding the inherent nutritional variation of corn is essential for broiler nutritionists to ensure corn-based diets provide consistent and efficient nutrient utilization to meet the high energy requirements of modern broiler strains [12].

In 2020, the largest corn producers globally were the United States (USA), Brazil (BRA), and Argentina (ARG) [13]. These countries played a key role in meeting the global demand for corn for animal feed [14]. To meet global demand for corn, vast transportation networks are required. During handling and transit, corn is exposed to various mechanical stresses that can compromise its physical integrity [15,16,17] and increase the content of broken corn [18], which is measured along with the percentage of foreign material as broken corn and foreign material (BCFM) to assess corn quality.

In the USA, both local and reimported corn play a critical role in broiler feed. The USA reimports corn for several reasons, despite being a major producer. In some regions, particularly near ports, producers may find it more economical to purchase reimported corn than to buy from domestic sources in the Heartland region [19], due to lower costs associated with shipping and tariffs [20]. Additionally, high-quality corn produced in the USA is often exported to meet demand in international markets, leaving a surplus that is sometimes reimported for use as cheap animal feed [21]. Another contributing factor is the variability in climate, both in the USA and abroad. Adverse weather conditions can lead to poor harvests, prompting the need for corn imports to ensure supply for the feed industry [22,23]. These factors combined create a dynamic market where corn flows across borders based on economic, environmental, and logistical considerations. However, increased handling, transportation, and prolonged storage can negatively impact grain quality, leading to a higher incidence of cracked kernels and a greater inclusion of foreign material [17].

Despite the widespread use of corn in poultry diets, variations in nutrient composition and digestibility due to geographic origin can significantly impact broiler performance, carcass yield, and feed efficiency [8]. However, limited data exists comparing the effects of corn from different origins on broiler performance and nutrient utilization. Previous work by our research group evaluated the effect of corn from the USA, BRA, and ARG on the growth performance and nutrient digestibility of broilers [24]. To expand on these findings and address the complexities of corn sourcing in the current market, the present study incorporated an additional variety, locally produced USA corn not subject to the re-importation process (USA-L). This extra comparison aims to provide a more comprehensive understanding of how differences in corn origin may influence broiler performance. In the earlier study, broilers fed with diets including USA and BRA corn showed improved FCR compared to broilers fed diets with corn from ARG. Breast meat weight was influenced by corn origin, with higher values observed in feeding broilers corn from BRA compared to ARG, although no differences were found between corn from BRA and USA. Building on these insights, the current study is a follow-up conducted two years after the initial evaluation, aiming to assess the influence of USA corn, both reimported (USA-R) and locally sourced without reimportation (USA-L), as well as South American corn sources (BRA and ARG), on broiler performance, nutrient digestibility, and processing yield from 1 to 35 d of age.

## 2. Materials and Methods

### 2.1. Animal Care

The poultry experiments described in this report were reviewed and sanctioned by the Institutional Animal Care and Use Committee at Auburn University (PRN 2022-5120).

### 2.2. Bird Husbandry

A total of 1200 male broiler chicks (YPM × Ross 708) were housed within a solid-walled house equipped with a negative pressure ventilation system, exhaust fans, air inlets, forced-air heaters, an evaporative cooling system, and an electronic temperature control. The chicks were randomly distributed into 48 floor pens (25 birds per pen; 0.12 m^2^ per bird) containing 5 nipple drinkers, a hanging pan feeder, and a clean surface layered with fresh litter. Feed and water were provided *ad libitum* throughout the trial. The lighting schedule was set to 23 h light and 1 h dark with a 4.0-foot candle (43 lux) intensity from d 1 to 25, and 20 h light and 4 h dark with an intensity of 0.50-foot candles (5 lux) from d 26 to 35. Room temperature was progressively reduced, starting at 33.0 °C on placement, then adjusted to 30.0 °C (d 4–7), 28.8 °C (d 8–14), 26.1 °C (d 15–21), 23.8 °C (d 22–28), and finally 20.0 °C (d 29–35). Bird welfare, mortality, temperature, humidity, and feed and water availability were monitored twice daily.

### 2.3. Feed Formulation, Manufacture, and Experimental Design

Three types of whole corn (USA-R, ARG, and BRA) were sourced from a single provider in Cartagena, Colombia, for use in the experimental diets described in this study. To minimize variability, all corn underwent identical shipping and storage conditions. These conditions included transportation in sealed containers and storage in a temperature- and humidity-controlled facility to prevent spoilage and to maintain consistency across treatments. Additionally, whole local corn from Alabama, USA (USA-L), routinely used at the Auburn University Feed Mill, served as the fourth corn source. Representative whole corn samples from each batch were collected using a manual stainless steel multi-level sampling probe and analyzed for nutritional composition using near-infrared spectroscopy (Model DS2500, FOSS NIR Systems, Silver Springs, MD, USA). Corn was ground using a 2-pair roller mill (Series 900-12, Roskamp Champion, CPM Inc., Chicago, IL, USA) with settings of 4-1.75 for starter feed and 4-1.90 for grower and finisher feeds (top-bottom roller pair). At the Charles C. Miller Jr. Poultry Research and Education Center, feed diets were prepared at the Auburn University Feed Mill. For 150 s (30 s dry mixing and 120 s wet mixing), ingredients were mixed using a twin-shaft mixer (Model 726, Scott Equipment Co., New Prague, MN, USA) to produce mash diets. These diets were then conditioned at 79.0 °C for 40 s and pelleted through a 4.0 mm die using a pellet mill (Model 1112-4, California Pellet Mill Co., Crawfordsville, IN, USA). Using ambient air, pellets were cooled in a counter-flow cooler (Model CC0909, California Pellet Mill Co., Crawfordsville, IN, USA), and starter diets were crumbled with a manual roll-adjustment crumbler (Model 624SS, same manufacturer).

Nutritional composition of the corn samples was used to formulate starter, grower, and finisher diets based on least-cost principles, adhering to Aviagen’s nutritional guidelines for Ross 708 male broilers [25] (Table 1 and Table 2). Diets were formulated to be isocaloric and isoproteic. Pens were randomly designated to one of the four experimental diets, resulting in each treatment having 12 replicate pens. Titanium dioxide (TiO_2_) was included in the finisher diets at 0.50% as an indigestible marker for nutrient digestibility analysis.

### 2.4. Measurements

Body weight (BW) and feed intake (FI) were measured on d 1, 10, 21, and 35 to calculate feed conversion ratio (FCR), determined by the division of FI by body weight gain (BWG), with adjustments made for mortality. All parameters were measured by pen.

Body weight (BW, g/bird) = ∑Weight of birds (g)/Number of birds

Feed intake (FI, g/bird) = Feed offered (g) − Residual feed (g)

Feed intake (FI, g/bird) = Feed intake (g)/Number of birds

Feed conversion ratio (FCR, g:g) = Feed intake (g)/Body weight gain (g) + Mortality weight (g)

Body weight gain (BWG, g/bird) = Final BW (g) − Initial BW (g)

On d 32, 10 birds per pen were randomly chosen and wing-banded for processing on d 36. Feed was withdrawn 10 h prior to processing, while water remained available for *ad libitum* consumption throughout the withdrawal phase. On d 36, birds were placed in coops and transported to Auburn University’s Fortenberry Processing Plant. Birds were mechanically processed through electrical stunning, exsanguination, scalding, plucking, and evisceration. In an ice bath, carcasses were chilled for 4 h, then drained on a rack for 5 min before weights were recorded. Afterwards, carcasses were deboned using stationary cones, and the weights of skinless breast fillets (pectoralis major), tenders (pectoralis minor), wings (drumette, wing portion, and wing tip), and legs (thigh and drumstick) were measured to determine part yields. Carcass yield was determined based on the live weight on d 36, while the yields of the breast fillets, tenders, wings, and legs were expressed as a percentage of the chilled carcass weight.

### 2.5. Nutrient Digestibility Analyses

Titanium dioxide was included in the finisher phase diets (22 to 35 d of age) to evaluate the apparent ileal digestibility (AID) of nutrients. On d 35, 5 birds from each pen were randomly chosen and euthanized via CO_2_ asphyxiation and cervical dislocation to gather ileal digesta and assess the digestibility of protein, energy, minerals, and fat. Ileal digesta was obtained by gently squeezing out the contents from 2 cm past the Meckel’s diverticulum until 2 cm before the ileal-cecal junction. Distilled water was used during this process to assist in the removal of the digesta. The collected ileal contents were pooled within each pen. After collection, the pooled ileal samples were kept on ice, transported to the laboratory, and stored at −20 °C until analysis. The samples were then lyophilized in a Virtis Genesis Pilot Lyophilizer (SP Industries, Warminster, PA, USA) and ground using an electric coffee grinder (Capresso 560.4 Infinity, Montvale, NJ, USA) on its finest setting. After grinding, the ileal digesta samples were submitted to a professional analytical facility (Dairy One Forage Laboratory, Ithaca, NY, USA) for the determination of crude protein (using AOAC Method 990.03), ether extract (analyzed per AOCS Procedure Am 5-04), and mineral content (following the methodologies outlined by Wolf et al. [26]). Titanium dioxide concentration in both the feed and ileal digesta was determined using the methods described by Short et al. [27]. Apparent ileal digestibility of crude protein, fat, and minerals was calculated using the following equation adapted from Stein et al. [28]:

$$Nutrient\;AID,\;(\%)=\left\{\frac{\left[{\left(\frac{Nutrient}{{TiO}_{2}}\right)}_{diet}-{\left(\frac{Nutrient}{{TiO}_{2}}\right)}_{digesta}\right]}{{\left(\frac{Nutrient}{{TiO}_{2}}\right)}_{diet}}\right\}\times 100$$

where $\left(\frac{Nutrient}{{TiO}_{2}}\right)$ represents the quotient of crude protein, fat, and minerals by the concentration of (TiO_2_) in either diet or ileal digesta.

### 2.6. Statistical Analyses

The data were analyzed using a complete randomized block design, where pen location was used as the blocking factor. Each treatment had 12 replications, with the pen serving as the experimental unit. A one-way ANOVA was performed using the GLM procedure in JMP PRO 15 [29]. Tukey’s HSD procedure was used to compare least square means among treatments. A *p*-value of ≤0.05 was considered statistically significant, while values between 0.0501 and 0.10 were interpreted as indicative of a trend.

## 3. Results

### 3.1. Chemical and Physical Analyses of Corn

Descriptive chemical analyses of corn are displayed in Table 3. Corn from USA-L had up to 0.5% more protein than corn from BRA, which had the lowest. Starch levels were highest in corn from BRA, about 3% more than corn from USA-L. Fat content remained consistent, with only a 0.13% difference between the highest and lowest values. Fiber differences were numerically minimal, with less than a 0.2% variation among samples.

Descriptive physical analyses of corn are presented in Table 4. Test weight was numerically similar among samples, with corn from BRA having the highest (749.14 kg/m^3^) and corn from USA-R the lowest (731.12 kg/m^3^). Moisture content varied by more than 1%, with corn from USA-L showing the highest (13.40%) and corn from BRA the lowest (12%). Damaged kernels were minimal across all treatments, though corn from USA-R had slightly more damage (0.50%) compared to the others. The amount of BCFM was highest in corn from USA-R (4.00%), approximately three times more than corn from USA-L, while corn from BRA had intermediate levels.

### 3.2. Broiler Performance

Growth performance results are summarized in Table 5. The incorporation of corn sourced from various origins had no effect (*p* > 0.05) on BW at 10, 21, and 35 d of age, nor on BWG from d 1 to 10, 1 to 21, and 1 to 35. From d 1 to 10, FI tended to be higher when broilers were fed USA-L and ARG corn compared to USA-R (*p* = 0.073). Feed intake was not affected (*p* > 0.05) by corn origin from d 1 to 21. However, from d 1 to 35, birds fed ARG and BRA corn showed a higher FI compared to birds fed USA-L corn (*p* = 0.012). Feed conversion ratio did not differ (*p* > 0.05) among broilers fed corn from different origins from d 1 to 10 and 1 to 21. However, from d 1 to 35, broilers fed diets containing corn from USA-L and USA-R had the lowest FCR, which were lower than those fed corn from BRA (*p* = 0.001).

### 3.3. Processing Yield

Processing yield results are summarized in Table 6. There was a tendency for broilers’ BW at d 36 (*p* = 0.075). Chilled carcass yield did not differ among treatments (*p* > 0.05). However, broilers fed diets containing ARG corn presented higher chilled carcass weight in contrast to broilers fed feed formulated with corn from USA-R (*p* = 0.032). The inclusion of corn from different origins had no effect (*p* > 0.05) on the weight or yield of the breast, tenders, wings, and legs.

### 3.4. Nutrient Digestibility

Nutrient digestibility results are summarized in Table 7. No differences (*p* > 0.05) were observed among dietary treatments for crude protein and crude fat digestibility. Broilers fed diets including corn from ARG exhibited higher phosphorus digestibility in comparison to those fed diets with corn from USA-L (*p* = 0.007). Calcium digestibility tended to be higher in broilers fed USA-L and USA-R corn (*p* = 0.097). Furthermore, potassium digestibility tended to be higher in birds fed diets containing corn from ARG (*p* = 0.096).

## 4. Discussion

The quality and nutritional value of corn can be significantly impacted by several factors, including physical damage and contamination with foreign material. Damaged kernels not only reduce grain quality but may also lower its digestibility and nutritional value [30]. Broken kernels can also increase the risk of mold infestation, which can further compromise protein, energy, and starch availability [31,32]. In particular, broken corn tends to have lower energy content compared to whole corn due to changes in its starch and protein structure [12], which can result in reduced digestibility and energy availability [33]. Foreign material, with its lower feeding and processing value and higher moisture content, poses a higher risk to quality deterioration during storage than broken corn [32]. Additionally, the portions of the corn kernel most prone to detachment and commonly found in BCFM are the tip cap and germ. The tip cap, located at the base of the kernel where it connects to the cob, is structurally weak and often breaks off during mechanical handling. The germ, although more firmly embedded, can also detach when kernels develop stress cracks, contributing to the fine material fraction during processing [34,35].

It is important to acknowledge that the corn samples used in this study represent only single batches from each origin, and obtaining these materials involved logistical complexity. While efforts were made to source commercially representative samples, these specific lots may not fully reflect the broader variability of corn exportation. Variations in nutrient composition can arise due to factors such as season, hybrid genetics, environmental conditions, and post-harvest handling practices, including storage and drying methods [36].

The reimportation of corn typically occurs when corn that was originally exported from the USA is brought back due to various market factors, such as changes in global demand or oversupply [19,37]. This process often involves corn that has been shipped abroad, and upon returning, it may include a higher percentage of BCFM [32]. The increase in BCFM is likely due to damage sustained during transportation and handling, which can degrade corn quality [17]. As a result, reimported corn generally contains more BCFM than domestic corn. The increase in BCFM can also influence storage and processing, as higher moisture content and the presence of contaminants can raise the risk of spoilage and mold development [32], especially since BCFM present a greater surface area compared to entire corn kernels, which can accelerate deterioration [35].

The results from this study indicate no impact of corn origin on BW and BWG at 10, 21, and 35 d of age. These findings align with previous studies, such as those by Melo-Durán et al. [38], who reported no differences in BW when broilers were fed diets containing different corn hybrids with similar nutrient composition (AME_n_: ≤ 304 kcal/kg; starch ≤ 3.8%; protein ≤ 1.95%; crude fiber ≤ 0.99%; fat ≤ 1.76%). Similarly, Vargas et al. [24] found no differences in BW during the experimental period when broilers were fed diets including corn from the USA, ARG, and BRA harvested in 2021. The current study, conducted two years later, supports these findings by showing that corn from different origins and harvest years had no significant impact on broiler growth performance.

Feed intake remained similar across treatments up to 21 d of age. However, from d 1 to 35, broilers fed diets containing corn from ARG and BRA showed higher FI compared to those fed corn from USA-L. Broiler-fed diets including USA-R corn exhibited no differences in FI compared to ARG, BRA, or USA-L. This increased FI suggests potential differences in kernel hardness and starch digestibility, as previously noted by Giacobbo et al. [39], who found that flint corn varieties often require higher FI due to reduced starch digestibility [40].

Feed conversion ratio was not affected during the initial growth phases; however, from 1 to 35 d, broilers fed corn from USA-L and USA-R exhibited a lower FCR than those fed corn from BRA. These results support findings by Brown et al. [41], who found that broilers fed dent corn diets exhibited improved FCR due to higher starch digestibility. Similarly, Moore et al. [42] highlighted that corn hybrids with lower kernel hardness tend to improve feed efficiency.

Stefanello et al. [33] reported that corn sourced from different regions within Brazil influenced energy and nutrient utilization in broilers, mainly due to variations in starch composition and digestibility. Specifically, corn from the North of Brazil exhibited higher AME and digestibility compared to corn from the South, likely due to differences in amylopectin content and fiber composition. Starch is the main component of corn, consisting of two glucose polymers: amylose and amylopectin. These molecules form concentric rings, with amylose occupying the spaces left by amylopectin during synthesis [43]. Amylose is a linear polysaccharide, whereas amylopectin is a branched polysaccharide; both forms of starch are polymers of α-D-glucose [44]. Amylopectin is more digestible than amylose due to its highly branched structure, which allows easier enzyme access. In contrast, amylose’s linear and compact nature slows down digestion, affecting glucose release and glycemic response [45]. Thus, the lower FCR observed in birds fed corn from USA-L and USA-R may be attributed to the softer endosperm structure, facilitating higher starch availability to enzymes [33].

The inclusion of corn from different origins had no influence on carcass characteristics. These findings are consistent with those of Giacobbo et al. [39], who reported no effects of corn hybrid inclusion on processing yields. Although no differences were observed in individual cut yields, broilers fed corn from ARG had a higher chilled carcass weight compared to those fed corn from USA-R. This suggests that while individual cuts remained proportionally similar, the overall carcass weight was greater, possibly due to subtle differences in energy utilization or carcass composition [46]. These results are supported by Clark et al. [30], who found that while different corn types did not affect carcass composition, variations in energy utilization could influence overall carcass weight. It is worth noting that these findings differ from those reported by Vargas et al. [24], who observed increased breast yield in broilers fed BRA corn compared to ARG corn.

No differences in digestibility of crude protein and crude fat were found among treatments, which contrasts studies by Kljak et al. [47] that reported that zein protein content, which is higher in flint corn, affects starch digestibility and overall nutrient absorption. However, phosphorus digestibility was higher in broilers fed corn from ARG compared to those fed corn from USA-L. This finding aligns with research by Cowieson et al. [3], who demonstrated that variations in phytate content among corn hybrids influence phosphorus retention in poultry diets. The inclusion of phytase in all dietary treatments likely contributed to the overall phosphorus digestibility observed across groups, as phytase hydrolyzes phytate to release bound phosphorus [48]. While phytase is generally effective, its efficacy can be influenced by the feed matrix, including differences in phytate content and structure among corn hybrids [49]. It is possible that ARG corn used presented a more accessible phytate substrate for phytase activity, though it was not directly evaluated. Additionally, higher FI observed in ARG-fed broilers may have contributed to improved nutrient digestibility, including phosphorus, through increased enzyme secretion or altered digesta transit time [50,51]. Therefore, the higher apparent phosphorus digestibility in the ARG group may reflect a combination of greater FI, differences in phytate accessibility, and consistent phytase activity across all diets.

The variation in the percentage of BCFM among corn samples may be associated with the high proportion of hard endosperm in South American corn varieties, such as those from ARG [52,53]. Flint corn is characterized by its hardness, resulting from a tightly packed starch-protein matrix, which can reduce starch digestibility and negatively affect broiler feed efficiency [39]. In contrast, corn from USA-L and USA-R, which is primarily dent corn, has a softer endosperm, facilitating higher starch digestion and nutrient absorption [41]. This difference in endosperm hardness likely explains the higher percentage of BCFM observed in USA corn samples compared to those from ARG and BRA in previous studies by Vargas et al. [24].

The presence of foreign material in poultry diets can significantly influence both nutrient digestibility and gut health. According to Abd El-Hack et al. [54], cob particles and fibrous plant material could potentially cause gastrointestinal damage. Fernandes et al. [55] also highlighted that ingestion of foreign material could alter the gut microbiota composition, which may lead to imbalances that affect feed conversion efficiency. The numerically higher percentage of foreign material in corn from USA-R could potentially pose a greater risk of microbial imbalance compared to the other treatments, such as corn from USA-L, where the percentage is lower. However, despite these differences in foreign material content, broiler growth performance remained unaffected across treatments.

Furthermore, contaminants like heavy metals, often found in foreign material, have been linked to oxidative stress and immune suppression in poultry [56]. This underscores the importance of minimizing such contaminants through stringent feed quality control measures. The corn from USA-R treatment showed a numerically higher percentage of BCFM compared to other treatments, which may contribute to increased oxidative stress and immune suppression, potentially impacting poultry health and performance. While these findings emphasize the importance of reducing foreign material to optimize feed quality and minimize potential health risks, it is important to note that broiler growth performance in this study remained similar across all treatments.

## 5. Conclusions

Broilers fed USA corn diets exhibited improved feed efficiency from 1 to 35 d of age, likely due to differences in starch digestibility. Carcass traits were unaffected by corn origin, except for higher chilled carcass weight in broilers fed ARG corn diets. Nutrient digestibility remained consistent, though phosphorus digestibility was higher in broilers fed corn from ARG, possibly due to differences in phytate levels. These findings emphasize the need to consider corn composition when formulating broiler diets, as variations in nutrient availability can impact feed utilization and overall performance. Future research should explore the underlying mechanisms driving differences in nutrient availability, including detailed analyses of starch structure, phytate content, and enzyme activity.

## Figures and Tables

**Table 1:** Composition of ingredients in the dietary treatments (% on an as-fed basis, unless otherwise specified) administered to YPM × Ross 708 male broilers from 1 to 35 d of age.

Ingredient	Starter 1 to 10 d				Grower 11 to 21 d				Finisher 22 to 35 d			
	USA-L ^1^	USA-R ^1^	ARG ^1^	BRA ^1^	USA-L	USA-R	ARG	BRA	USA-L	USA-R	ARG	BRA
Corn	53.12	53.34	53.74	54.13	58.50	58.74	59.16	59.57	61.59	61.84	62.30	62.73
Soybean meal, 48% CP	40.11	40.08	40.02	39.96	35.27	35.25	35.20	35.16	31.71	31.67	31.62	31.57
Poultry oil	3.11	2.92	2.58	2.25	3.34	3.13	2.75	2.39	3.77	3.54	3.14	2.76
Dicalcium phosphate, 18% P	1.04	1.04	1.04	1.04	0.66	0.66	0.66	0.66	0.37	0.37	0.37	0.37
Limestone	0.89	0.89	0.89	0.89	0.67	0.67	0.67	0.67	0.62	0.62	0.62	0.63
Salt	0.50	0.50	0.50	0.50	0.50	0.50	0.50	0.50	0.50	0.50	0.50	0.50
DL-Methionine, 99%	0.42	0.42	0.42	0.42	0.37	0.37	0.37	0.37	0.34	0.34	0.34	0.34
L-Lysine HCl	0.27	0.27	0.27	0.27	0.22	0.22	0.22	0.23	0.20	0.20	0.20	0.20
L-Threonine, 98%	0.16	0.16	0.16	0.16	0.13	0.13	0.13	0.13	0.11	0.10	0.10	0.10
L-Valine	0.08	0.07	0.07	0.07	0.06	0.06	0.06	0.06	0.05	0.05	0.05	0.04
L-Isoleucine	0.03	0.03	0.03	0.03	0.03	0.03	0.03	0.03	0.04	0.04	0.04	0.04
Choline Cl, 70%	0.06	0.06	0.06	0.06	0.06	0.06	0.06	0.06	0.06	0.06	0.06	0.06
Trace-mineral premix ^2^	0.10	0.10	0.10	0.10	0.10	0.10	0.10	0.10	0.10	0.10	0.10	0.10
Vitamin premix ^3^	0.10	0.10	0.10	0.10	0.08	0.08	0.08	0.08	0.05	0.05	0.05	0.05
Titanium dioxide	-	-	-	-	-	-	-	-	0.50	0.50	0.50	0.50
OptiPhos^®^ Plus ^4^, g/kg	0.01	0.01	0.01	0.01	0.01	0.01	0.01	0.01	0.01	0.01	0.01	0.01

^1^ Diets formulated with corn produced in the USA locally (USA-L) and USA reimported (USA-R), Argentina (ARG), and Brazil (BRA). ^2^ Mineral premix includes per kg of diet: Mn (manganese sulfate), 120 mg; Zn (zinc sulfate), 100 mg; Fe (iron sulfate monohydrate), 30 mg; Cu (tri-basic Cu chloride), 8 mg; I (ethylenediaminedihydroxide), 1.4 mg; and Se (sodium selenite), 0.3 mg. ^3^ Vitamin premix includes per kg of diet: Vitamin A (Vitamin A acetate), 187,390 IU; Vitamin D (cholecalciferol), 6614 IU; Vitamin E (DL-alpha tocopherol acetate), 66 IU; menadione (menadione sodium bisulfate complex), 4 mg; Vitamin B12 (cyanocobalamin), 0.03 mg; folacin (folic acid), 2.6 mg; D-pantothenic acid (calcium pantothenate), 31 mg; riboflavin (riboflavin), 22 mg; niacin (niacinamide), 88 mg; thiamin (thiamin mononitrate), 5.5 mg; D-biotin (biotin), 0.18 mg; and pyridoxine (pyridoxine hydrochloride), 7.7 mg. ^4^ OptiPhos^®^ Plus (Huvepharma Inc., Peachtree City, GA, USA) provided 1000 FTU/kg of phytase activity per kg of diet.

**Table 2:** Nutrient composition (% as-fed basis, unless otherwise noted) of diets fed to YPM × Ross 708 male broilers from 1 to 35 d of age.

Calculated Analysis	Starter				Grower				Finisher			
	1 to 10 d				11 to 21 d				22 to 35 d			
	USA-L ^1^	USA-R ^1^	ARG ^1^	BRA ^1^	USA-L	USA-R	ARG	BRA	USA-L	USA-R	ARG	BRA
AME_n_^2^, kcal/kg	2975	2975	2975	2975	3050	3050	3050	3050	3100	3100	3100	3100
Crude protein	23.80	23.60	23.72	23.73	21.77	21.56	21.69	21.72	20.22	20.00	20.13	20.16
Digestible Lys	1.32	1.32	1.32	1.32	1.18	1.18	1.18	1.18	1.08	1.08	1.08	1.08
Digestible Met	0.71	0.71	0.71	0.71	0.65	0.65	0.65	0.65	0.60	0.60	0.60	0.60
Digestible TSAA ^3^	1.00	1.00	1.00	1.00	0.92	0.92	0.92	0.92	0.86	0.86	0.86	0.86
Digestible Thr	0.88	0.88	0.88	0.88	0.79	0.79	0.79	0.79	0.72	0.72	0.72	0.72
Digestible Val	1.00	1.00	1.00	1.00	0.91	0.91	0.91	0.91	0.84	0.84	0.84	0.84
Digestible Ile	0.88	0.88	0.88	0.88	0.80	0.80	0.80	0.80	0.75	0.75	0.75	0.75
Digestible Leu	1.65	1.65	1.66	1.66	1.55	1.55	1.55	1.55	1.46	1.46	1.47	1.47
Digestible Arg	1.40	1.40	1.40	1.40	1.27	1.27	1.27	1.27	1.17	1.17	1.17	1.17
Digestible Trp	0.26	0.26	0.26	0.26	0.23	0.23	0.23	0.23	0.21	0.21	0.21	0.21
Total calcium	0.95	0.95	0.95	0.95	0.75	0.75	0.75	0.75	0.65	0.65	0.65	0.65
Non-phytate phosphorus	0.50	0.50	0.50	0.50	0.42	0.42	0.42	0.42	0.36	0.36	0.36	0.36
Sodium	0.20	0.20	0.20	0.20	0.20	0.20	0.20	0.20	0.20	0.20	0.20	0.20
Chloride	0.30	0.30	0.30	0.30	0.30	0.30	0.30	0.30	0.30	0.30	0.30	0.30
Choline, mg/kg	1700	1700	1700	1700	1600	1600	1600	1600	1500	1500	1500	1500

^1^ Diets formulated with corn produced in the USA locally (USA-L) and USA reimported (USA-R), Argentina (ARG), and Brazil (BRA). ^2^ AME_n_ = nitrogen-corrected apparent metabolizable energy. ^3^ TSAA = total sulfur amino acids.

**Table 3:** Chemical analysis (%) of corn from different origins.

Treatment ^1^	Crude Protein	Starch	Fat	Crude Fiber
USA-L	7.99	62.32	3.66	1.78
USA-R	7.74	63.98	3.56	1.86
ARG	7.81	64.37	3.67	1.77
BRA	7.49	65.28	3.54	1.74

^1^ Diets formulated with corn produced in the USA locally (USA-L) and USA reimported (USA-R), Argentina (ARG), and Brazil (BRA).

**Table 4:** Physical analysis of corn from different origins.

Treatment ^1^	Test Weight (kg/m^3^)	Moisture (%)	Damaged Kernels (%)	Broken Corn and Foreign Material (%)
USA-L	746.57	13.40	0.20	1.30
USA-R	731.12	13.10	0.50	4.00
ARG	733.70	12.60	0.00	3.40
BRA	749.14	12.00	0.20	2.60

^1^ Diets formulated with corn produced in the USA locally (USA-L) and USA reimported (USA-R), Argentina (ARG), and Brazil (BRA).

**Table 5:** Effect of corn origin on growth performance of YPM × Ross 708 male broilers from 1 to 35 d of age.

Treatment ^5^	BW ^1^, g/bird				BWG ^2^, g/bird			FI ^3^, g/bird			FCR ^4^, g:g		
	1 d	10 d	21 d	35 d	1–10 d	1–21 d	1–35 d	1–10 d	1–21 d	1–35 d	1–10 d	1–21 d	1–35 d
USA-L	39	276	998	2445	237	959	2406	276	1219	3372 ^b^	0.996	1.223	1.376 ^c^
USA-R	39	270	984	2438	231	945	2399	269	1196	3396 ^ab^	0.999	1.214	1.385 ^bc^
ARG	39	275	993	2488	236	954	2449	275	1218	3470 ^a^	1.000	1.223	1.397 ^ab^
BRA	39	271	992	2462	232	953	2422	273	1222	3463 ^a^	1.011	1.226	1.401 ^a^
SEM ^6^	0.19	2	9	19	2	9	19	2	9	24	0.005	0.004	0.004
*p*-value	0.392	0.153	0.731	0.263	0.153	0.730	0.260	0.073	0.140	0.012	0.115	0.141	<0.001

^a–c^ Least square means within a column with different superscripts differ significantly (*p* ≤ 0.05). Least-square means of 12 replicate pens, with 25 birds each. ^1^ Body weight. ^2^ Body weight gain. ^3^ Feed intake. ^4^ Feed conversion ratio corrected for mortality. ^5^ Diets formulated with corn produced in the USA locally (USA-L) and USA reimported (USA-R), Argentina (ARG), and Brazil (BRA). ^6^ Standard error of the mean.

**Table 6:** Processing yields of YPM × Ross 708 male broilers fed diets containing corn from different origins from 1 to 35 d of age.

Treatment ^3^	d 36 Live Weight	Chilled Carcass		Breast		Tenders		Wings		Legs	
	g/bird	Weight, g/bird	Yield ^1^, %	Weight, g/bird	Yield ^2^, %	Weight, g/bird	Yield ^2^, %	Weight, g/bird	Yield ^2^, %	Weight, g/bird	Yield ^2^, %
USA-L	2589	1941 ^ab^	75.33	561	28.76	118	6.05	195	10.01	552	28.42
USA-R	2572	1911 ^b^	74.54	556	28.97	118	6.16	191	9.95	544	28.23
ARG	2603	1971 ^a^	75.61	568	28.91	120	6.11	196	9.99	555	28.18
BRA	2590	1957 ^ab^	75.41	570	29.11	118	6.06	195	9.99	559	28.4
SEM ^4^	9	15	0.54	6	0.17	1	0.05	1	0.05	4	0.12
*p*-value	0.075	0.032	0.516	0.346	0.537	0.474	0.430	0.057	0.893	0.076	0.410

^a,b^ Least square means within a column with different superscripts differ significantly (*p* ≤ 0.05). Least-square means of 12 replicate pens, with 25 birds each. ^1^ At 36 d of age, chilled carcass yield was expressed as a percentage of live weight. ^2^ Yields presented as a percentage of the cold carcass weight. ^3^ Diets formulated with corn produced in the USA locally (USA-L) and USA reimported (USA-R), Argentina (ARG), and Brazil (BRA). ^4^ Standard error of the mean.

**Table 7:** Apparent ileal nutrient digestibility of YPM × Ross 708 male broilers fed diets including corn from distinct origins from 22 to 35 d of age.

Treatment ^2^	Apparent Ileal Digestibility ^1^, %				
	Crude Protein	Crude Fat	Phosphorus	Calcium	Potassium
USA-L	81.47	88.32	75.13 ^b^	64.03	89.90
USA-R	81.70	88.02	78.07 ^ab^	66.20	90.77
ARG	83.33	89.99	79.92 ^a^	63.72	90.97
BRA	81.99	88.60	77.95 ^ab^	61.38	89.77
SEM ^3^	0.57	1.00	0.90	1.00	0.40
*p*-value	0.125	0.564	0.007	0.097	0.096

^a,b^ Least square means within a column with different superscripts differ significantly (*p* ≤ 0.05). ^1^ Least-square means of 12 replicate pens, with 25 birds each. ^2^ Diets formulated with corn produced in the USA locally (USA-L) and USA reimported (USA-R), Argentina (ARG), and Brazil (BRA). ^3^ Standard error of the mean.

## Data Availability

All data are contained within the article.
